# Digital pathways to recovery: Enhancing mobility and well-being post-TBI

**DOI:** 10.6026/973206300211960

**Published:** 2025-07-31

**Authors:** Athya Farheen Mohammed Sulthan, Raghavendran R., Shankar Shanmugam Rajendran, Duraikannu Anandhi, Padmavathy Murugan, Pradeepa Govind, Vasanth Pandian Thommai Antony Savari Muthu

**Affiliations:** 1 Department of Medical Surgical Nursing, College of Nursing, Madras Medical College, Chennai, Tamil Nadu, India; 2 Institute of Neurosurgery, MMC & RGGGH, Chennai, Tamil Nadu, India; 3 Department of Child Health Nursing, College of Nursing, Madras Medical College, Chennai, Tamil Nadu, India

**Keywords:** Digital health intervention, head injury, physical ability, life-style modification, sense of coherence, rehabilitation nursing

## Abstract

Traumatic brain injury (TBI) leads to significant physical and psychological challenges, including impaired mobility and a disrupted sense of coherence. Traditional rehabilitation faces obstacles such as limited access, cost and poor adherence. A mixed-methods study at Rajiv Gandhi Government General Hospital explored the explored the physical mobility challenges and impact of TBI patients. The intervention group showed significant improvements in physical, lifestyle and emotional outcomes. Thus, digital health interventions can enhance TBI rehabilitation by improving physical abilities, lifestyle adaptation and emotional resilience.

## Background:

As traumatic brain injury (TBI) rates continue to rise globally due to road accidents, falls and violence, healthcare systems are increasingly called to address not only the physical aftermath but also the broader psycho-social implications [1]. Patients recovering from TBI often face profound physical mobility challenges, disruptions in lifestyle and emotional struggles that compromise their independence, self-worth and overall coherence in life. These consequences extend far beyond the initial trauma, affecting long-term recovery and quality of life [2]. Central to this recovery is the concept of sense of coherence, a person's ability to perceive life as comprehensible, manageable and meaningful. Alongside physical rehabilitation, promoting this psychological resilience is vital to help TBI survivors regain stability and purpose. However, conventional rehabilitation services frequently encounter limitations in accessibility, continuity and personalised care [3]. In this context, digital health interventions are emerging as transformative tools in neuro-rehabilitation. By leveraging mobile applications, video-based education and tele consultations, these interventions offer scalable, patient-tailored support that can extend care beyond hospital walls [4]. They not only enable consistent monitoring and guided therapy but also encourage self-care and active engagement in the healing process. Despite growing evidence in other domains, the role of digital health interventions in improving physical ability, lifestyle behaviours and sense of coherence, specifically among post-head injury patients, remains underexplored [5]. Therefore, it is of interest to bridge that gap by evaluating the impact of a structured digital health intervention on physical and psychological outcomes in TBI patients at a tertiary care hospital in Chennai, India.

## Methodology:

An exploratory sequential mixed-method (Qual-Quan) design was utilised to determine the impact of a digital health intervention on physical function, lifestyle habits and sense of coherence among individuals with post-head injury. The research was carried out in the Neurosurgery Outpatient Department of Rajiv Gandhi Government General Hospital (RGGGH), Chennai, after obtaining ethical approval from the Institutional Ethics Committee of Madras Medical College. During qualitative phase, five participants were selected through purposive sampling and interviewed using an unstructured tool to explore issues related to mobility and coping strategies. Themes generated from this phase were analysed using thematic content analysis and guided the development of the digital intervention. In the quantitative phase, 60 participants were chosen via non-probability convenience sampling and evenly assigned to experimental and control groups. Inclusion criteria were adults with a head injury for more than three months who were willing and able to participate. Exclusion criteria included cognitive or psychiatric illness. The intervention group received a 30-day digital program with video rehab, mobile guidance and self-care prompts, while the control group received routine care. Tools included the Barthel Index, Lifestyle Questionnaire and SOC-13 Scale. Analysis was done using SPSS.

## Results:

Participants were predominantly male (66.67%), aged 31-50 years (58.33%), with secondary education (71.67%) and a history of road traffic accidents (76.67%). Both groups were comparable at baseline, with no significant differences. NVivo-based thematic analysis revealed five key themes: loss of independence, frustration during daily tasks, emotional withdrawal, self-motivation and digital support as a motivator. These findings supported the quantitative outcomes, highlighting how digital tools enhanced autonomy and coping. Pre-test scores were similar. Post-intervention, 73.33% in the experimental group showed improved Barthel Index scores, shifting from moderate to high independence. The control group had minimal change, with 63.33% remaining moderate (P < 0.05) shown in the Table 1 (see PDF). At baseline, both groups showed poor lifestyle adherence. After intervention, 76.67% of the experimental group moved to high scores, while the control group remained large unchanged (P < 0.05) show in Table 2 (see PDF). Baseline SOC levels were moderate in both groups. Post-test, 80.00% in the experimental group reached high SOC, while the control group showed no major improvement (P < 0.05) shown in Table 3 (see PDF). A positive correlation (r = 0.41, P = 0.001) was found between physical ability and SOC. Lifestyle modification also showed positive links with both. Better outcomes were noted in participants aged 31-40, those with no comorbidities, higher education and urban backgrounds. These trends were more evident in the experimental group, though not statistically significant in controls.

## Discussion:

The present study found that digital health interventions significantly improved physical ability, lifestyle modification and sense of coherence among post-head injury patients. These findings are consistent with previous work, including the study by Karvandi *et al.* (2024) [5], which highlighted the role of remote digital tools in addressing persistent symptoms of mild TBI and supporting continuous recovery. Their work emphasised the importance of accessible, patient-centred interventions to enhance physical and emotional outcomes. The positive correlation between physical ability and sense of coherence (r = 0.41, P = 0.001) in this study underscores the link between improved functional independence and psychological adaptation. These results are also supported by Malathi Dhakshnamoorthy, *et al.* (2025) [6], who found a strong positive relationship (r = 0.74, p < 0.05) between perceived social support and psychological well-being among caregivers of TBI patients. These results are in line with findings from Juengst *et al.* (2019) [7] and Avramovic *et al.* (2023) [8], who demonstrated that mobile health solutions foster autonomy, emotional resilience and daily engagement among individuals with acquired brain injuries. The thematic insights, such as regaining control, overcoming frustration and viewing digital guidance as empowering, further validate the intervention's impact beyond measurable scores. Together, these outcomes suggest that integrating digital tools into rehabilitation nursing practice may bridge existing care gaps, especially for patients with limited access to traditional services. As a non-invasive, scalable and cost-effective strategy, digital health intervention offers promising potential for improving holistic recovery in TBI care [9, 10]. Future research should examine long-term adherence, customisation and outcomes across diverse patient populations.

## Conclusion:

Digital health interventions effectively enhanced physical ability, lifestyle modification and sense of coherence among post-head injury patients. It empowered individuals through accessible, personalised care and supported emotional resilience. Integrating such technology into rehabilitation can promote holistic recovery and improve quality of life in neuro-nursing practice.
